# Inversely 3D-Printed β-TCP Scaffolds for Bone Replacement

**DOI:** 10.3390/ma12203417

**Published:** 2019-10-18

**Authors:** Michael Seidenstuecker, Svenja Lange, Steffen Esslinger, Sergio H. Latorre, Rumen Krastev, Rainer Gadow, Hermann O. Mayr, Anke Bernstein

**Affiliations:** 1 G.E.R.N. Tissue Replacement, Regeneration & Neogenesis, Department of Orthopedics and Trauma Surgery, Medical Center - Albert-Ludwigs-University of Freiburg, Faculty of Medicine, Albert-Ludwigs-University of Freiburg, Hugstetter Straße 55, 79106 Freiburg, Germany; svenja-lange95@web.de (S.L.); sergio.latorre@uniklinik-freiburg.de (S.H.L.); hermann.mayr@uniklinik-freiburg.de (H.O.M.); anke.bernstein@uniklinik-freiburg.de (A.B.); 2 Faculty of Applied Chemistry, Reutlingen University, Alteburgstraße 150, 72762 Reutlingen, Germany; Rumen.Krastev@reutlingen-university.de; 3 Institute for Manufacturing Technologies of Ceramic Components and Composites (IMTCCC), Faculty 07, University of Stuttgart, Allmandring 7b, 70569 Stuttgart, Germany; Steffen.Esslinger@gsame.uni-stuttgart.de (S.E.); rainer.gadow@ifkb.uni-stuttgart.de (R.G.); 4 GSaME – Graduate School of Excellence advanced Manufacturing Engineering, University of Stuttgart, Nobelstraße 12, 70569 Stuttgart, Germany

**Keywords:** FDM, inversely, β-TCP, bone replacement

## Abstract

The aim of this study was to predefine the pore structure of β-tricalcium phosphate (β-TCP) scaffolds with different macro pore sizes (500, 750, and 1000 µm), to characterize β-TCP scaffolds, and to investigate the growth behavior of cells within these scaffolds. The lead structures for directional bone growth (sacrificial structures) were produced from polylactide (PLA) using the fused deposition modeling techniques. The molds were then filled with β-TCP slurry and sintered at 1250 °C, whereby the lead structures (voids) were burnt out. The scaffolds were mechanically characterized (native and after incubation in simulated body fluid (SBF) for 28 d). In addition, biocompatibility was investigated by live/dead, cell proliferation and lactate dehydrogenase assays. The scaffolds with a strand spacing of 500 µm showed the highest compressive strength, both untreated (3.4 ± 0.2 MPa) and treated with simulated body fluid (2.8 ± 0.2 MPa). The simulated body fluid reduced the stability of the samples to 82% (500), 62% (750) and 56% (1000). The strand spacing and the powder properties of the samples were decisive factors for stability. The fact that β-TCP is a biocompatible material is confirmed by the experiments. No lactate dehydrogenase activity of the cells was measured, which means that no cytotoxicity of the material could be detected. In addition, the proliferation rate of all three sizes increased steadily over the test days until saturation. The cells were largely adhered to or within the scaffolds and did not migrate through the scaffolds to the bottom of the cell culture plate. The cells showed increased growth, not only on the outer surface (e.g., 500: 36 ± 33 vital cells/mm² after three days, 180 ± 33 cells/mm² after seven days, and 308 ± 69 cells/mm² after 10 days), but also on the inner surface of the samples (e.g., 750: 49 ± 17 vital cells/mm² after three days, 200 ± 84 cells/mm² after seven days, and 218 ± 99 living cells/mm² after 10 days). This means that the inverse 3D printing method is very suitable for the presetting of the pore structure and for the ingrowth of the cells. The experiments on which this work is based have shown that the fused deposition modeling process with subsequent slip casting and sintering is well suited for the production of scaffolds for bone replacement.

## 1. Introduction

Bone disorders and defects continue to increase in our society. The reason for this is the increasing average age of the population [1]. According to the Federal Statistical Office, 191,272 endoprostheses were implanted in knee joints in Germany in 2017 [2]. The development of 3D printing and the associated tissue engineering has opened up new possibilities for bone replacement [3]. Bone tissue engineering is a very complicated area [1]. Its difficulty lies in producing complex structures on a scale necessary for human application [4]. These scaffolds are usually made of biodegradable materials with different porosities [1,5]. Above all, they provide mechanical support during the repair or regeneration processes of the bone. A further successful development has been the combination of scaffolds with embedded growth factors and medications, both of which cause an increased cell growth of bone cells and thus a faster healing of defects [1]. Since the biomechanical systems of bones is complex, scaffolds used to treat bone defects must meet certain requirements: Biocompatibility, mechanical properties, and pore size [6].

There are different types of 3D printing. On the one hand there is powder based 3D-printing (which we have already described in a previous paper [7]), in which a binder is printed into a powder bed. Another possibility is selective laser sintering (SLS), in which powder is selectively melted by means of a laser [8]. The limiting factor regarding the size of the constructs in this case is the particle size of the powder. Another method that should be mentioned is 3D plotting, in which a ceramic cement, e.g., oil-based cement, is printed into an aqueous solution. When it comes into contact with water, the setting reaction begins, and the construct solidifies [9]. Another of these 3D printing processes is the fused deposition modeling (FDM) process, which was first described by S. Scott Crump [10] in the late 1980s and has since become indispensable in the research and development of 3D printing. The FDM process is a typical approach that uses heat to produce ceramic scaffolds [6,11]. The advantage of the FDM method is that it can produce almost unlimited complex geometric structures [12,13]. The ceramic model is produced with the help of a framework made of a thermoplastic material, in this case polylactide (PLA). The framework provides an inverse shape and structure for the subsequent scaffold. This means that the PLA is used as a “sacrificial template” into which the ceramic, in our case β-TCP, is cast [14]. 

The basis for the 3D printing of the PLA scaffold is a computer aided design (CAD) drawing, which is then printed using the FDM process. Calcium phosphates (CaP) is mainly used for biomedical applications [15]. CaP is non-toxic and does not cause a foreign body reaction. These properties make CaP a biocompatible and therefore very interesting biomaterial [6,12,16]. In all previous studies, only 3D scaffolds were printed, into which bone then arbitrarily grows; as such, the inverse printing stands in the foreground in this study. The aim of the project was to define the pore structure within a 3D construct. This means that the areas into which the bone grows are defined as porous guide rails. The FDM method was very well suited for this purpose, as the guide rails for directional bone growth could then be printed as sacrificial structures in PLA. PLA was used because it is a biomaterial and burns off at low temperatures (compared to the sinter temperatures of CaP). Finally, the β-TCP scaffolds with preset pore structure are characterized in this paper, and the cell growth behavior within the scaffolds depending on the size of the voids (sacrificial structures) are investigated.

## 2. Materials and Methods

### 2.1. Sample Manufacturing

The ceramic scaffolds were designed and manufactured at the University of Stuttgart at the Institute for Manufacturing Technology of Ceramic Components and Composites. The manufacturing principle was based on a three-dimensional printed scaffold. The FDM process was used to print a PLA form using a Prusa i3 MK3 3D printer (Prusa Research, Prague, Czech Republic). This form represented the negative (inner pore structure) of the later ceramic scaffold. To produce the ceramic model, the ceramic was cast into the plastic mold using the slip casting process. For the PLA molds with strand widths of 500 and 1000 µm, a 0.5 mm nozzle was used. A 0.4 mm nozzle was used for printing the PLA molds with a strand width of 750 µm. The diameter of the commercially available filament was 1.75 mm. To avoid the warping of the printed structures, the print bed was heated up to 60 °C, which was near the glass transition point of the used PLA. The nozzle temperature was 215 °C. The layer thickness was 250 µm for every model.

To obtain the final bioceramic scaffolds, a water-based slurry was filled into the PLA molds. The slurry contained 70 wt% of β-TCP and 1 wt% based on solid content DOLAPIX CE64 (Zschimmer & Schwarz, Lahnstein, Germany) as a dispersant. The grain sizes of the β-TCP powder ranged from 0.6 to 40 µm (d10 = 2.0 ± 0.04 µm; d50 = 5.27 ± 0.08 µm, and d90 = 14.84 ± 0.09 µm).

For the slurry infill, the molds stood on a porous plaster plate because they were used for the conventional slip casting process. The water was removed by capillary forces, and the remaining ceramic particles were slightly compacted during this process. As a result, the ceramics became denser and increasingly solid [17]. After drying for 24 h on the plaster plate, the sacrificial PLA molds were burnt out at about 450 °C for 4 h. Only the ceramic with defined pore sizes remained. In order to give the model mechanical strength and density, the samples were sintered at the end. The temperature was heated up to 1000 °C at 125 K/h and then to 1250 °C at 100 K/h. This temperature was maintained for four hours and then cooled down again with the same temperature steps [17,18]. Samples with strand widths of 500, 750, and 1000 µm were produced with the 3D printer, filled with ceramic slurry, and sintered according to the above protocol. In the following, the designations 500, 750, and 1000 µm remained, which herein refer to the empty spaces between the β-TCP strands for the differentiation of the samples. 

### 2.2. Sample Characterization

#### 2.2.1. Weight and Dimensions

The β-TCP scaffolds were weighed and measured. An Olympus SZ61 stereomicroscope (Olympus, Shinjuku, Japan) was used to measure strand width and pore size. The voids were then determined using Image-J (Fiji Version 1.52h). The sample dimensions were measured with a Burgwächter PS 7215 digital caliper gauge (Burg-Wächter, Wetter-Volmarstein, Germany). The scaffolds were weighed with a Sartorius Practum analytical balance (Sartorius, Goettingen, Germany).

#### 2.2.2. Surface Roughness

Surface roughness was measured using KEYENCE’s VKX-210 3D laser scanning microscope (Keyence, Osaka, Japan). The images were taken with 400× magnification. The measurements were taken at room temperature. Three samples of each size and five different positions were measured on the scaffolds (n = 15). The surface roughness was calculated using the KEYENCE VK Analysis Module (V3.5.0.0).

#### 2.2.3. Porosimetry

As the micro pores within the ceramic are exclusively the responsibility of the ceramic slurry and not the macro porosity specified by FDM, new specimens without macro pores were produced for this test and examined by mercury porosimetry. Within the scope of this work, the porosity (micro pores) of the ceramic scaffolds was measured with a Porotec 140/440 porosimeter (Porotec GmbH, Hofheim, Germany) at the University of Stuttgart. In order to make sure that there was no water left in the specimens, they were annealed for 24 h at 105 °C. For the determination of pore sizes with a diameter of 5.8–1.4 µm a Pascal 140 low pressure porosimeter was used (pressure was built-up up to 0.1 KPa). Afterwards, the specimens were transferred into a Pascal 440 high pressure porosimeter (pressure was built-up up to 400 MPa) for the measurement of the pore sizes from 5.8 to 1.8 nm.

#### 2.2.4. Mechanical Strength

The compression test was carried out on a Zwick Z005 universal testing machine (Zwick Roell, Ulm, Germany). For this, 10 untreated and 10 simulated body fluid (SBF)-treated scaffolds (see 2.4.) of each size were used. The samples were centrally placed on the sample plate and then subjected to a preload of 1 N. The force F_B_ up to the break of the scaffolds and the maximum force F_max_ applied to the specimen were then determined.

#### 2.2.5. Microstructure and Elemental Analysis

To investigate the pore structure within the ceramic, images were taken with an ESEM FEI QUANTA 250 FEG (FEI, Hilsboro, OR, USA) with an Oxdord EDX (energy dispersive X-ray spectrometer) unit (Oxford Instruments, Tubney Woods, UK). The samples were glued to the pin sample holders with a double-sided carbon guide pad and fixed in the microscope in the sample holder. Subsequently, images were taken with an excitation voltage of 10 kV and the HFW (horizontal field width) magnifications of 1.49, 373, 93.3, and 23.3 µm. For the elemental analysis using EDX, the samples were cut through the middle using a razor blade. Work was performed at room temperature, with a 5 min dead time corrected measurement and an excitation voltage of 20 kV.

### 2.3. Biocompatibility

All experiments were performed with MG-63 cells (ATCC CRL 1427). The cells were first thawed from the liquid nitrogen tank (at −196 °C) in passage 15 and cultured in a Dulbecco’s Modified Eagle Medium (DMEM) with an F12 nutrient content and additives 1% penicillin/streptomycin (P/S) and 10% fetal bovine serum (FBS) in a New Brunswick Galaxy 170R incubator (Eppendorf, Hamburg, Germany) at 37 °C, with a CO_2_ saturation of 5%. The cells were passed twice a week and then split 1:10 and 1:5. For all biocompatibility tests and experiments with SBF, the scaffolds were heat sterilized at 200 °C for 4 h in a drying oven UF500 (Memmert, Schwabach, Germany).

#### 2.3.1. Live/Dead Assay

The live/dead examinations were performed after 3, 7, and 10 days. Three samples per scaffold size were placed in cell culture plates. Subsequently, 50,000 cells each, which were in 200 µL of medium, were placed directly on the samples and incubated for 2 h at 37 °C with a CO_2_ saturation of 5% in the incubator so that the cells could adhere to the surface of the samples. After two hours, 2.5 mL of a DMEM-F12 (Art. No. BE12-719F, Lonza, Basel, Switzerland) complete medium was added to each well and incubated in the incubator for a defined time (3, 7, 10 days). After this time, the sample was prepared for staining. The staining solution was first prepared by adding 2 mL of DPBS (Art. No. 14190-094, Gibco, Grand Island, NE, USA) to a Falcon and 4 µL of Ethidium Homodimer III (Eth D-III) solution. The solution was then mixed. Then, 1 µL of calcein dye was added and mixed again. Finally, the prepared solution had to be covered with aluminium foil due to the sensitive fluorescent dye. For staining after the first cultivation, the medium was removed and the cells were washed to eliminate serum esterase activity. Subsequently, the cells were stained according to the protocol [19]. After incubation, the cells were inspected under a fluorescence microscope. For evaluation, images were taken with an Olympus fluorescence microscope (BX51) from five different positions with 5× and 10× magnification on the scaffolds. The specimens were then cut vertically with a razor blade and recorded at three different locations with the 5× and 10× magnifications. Then, the ceramics were cut horizontally and viewed at the same three positions with the known magnifications. Living cells fluoresce green under blue light, and dead cells fluoresce red.

#### 2.3.2. Cell Proliferation Assay

For the experiment, three samples of each of the differently sized scaffolds were examined after 3, 7 and 10 days using the WST-1 test. A Nunc™ Thermanox™ Coverslip (Thermo Fisher Scientific) membrane served as positive control. All samples and controls were equally covered with 50,000 cells in 200 µL. The cells were incubated for 2 h at 37 °C with a CO_2_ saturation of 5% in the incubator so that they could adhere to the surface of the sample. At the end of this period, 2.5 mL of the DMEM-F12 complete medium was added to each sample and incubated. A medium change with the DMEM-F12 with the 10% FBS and 1% P/S additives was performed for days 7 and 10. The plate from day 3 was prepared for the WST evaluation. The medium was aspirated, and the wells were washed three times with PBS. The samples and the Thermanox Coverslips were then transferred to a new well, and then 2.5 mL of the DMEM-F12 phenol red free (Art. No. 11039-021, Gibco, Grand Island, NE, USA) with the 1% P/S and 1% FBS additives were added to the wells with the sample (TCP + R). Four-hundred microliters of the medium were added to the previously used empty Sample Wells (TCP), Positive Control (C + R), Empty Control Well (C+), and Blank. The blank contained only the DMEM medium without phenol red and was measured to account for background absorption. Ten percent WST reagent (Art. No. 05015944001, Roche, Basel, Switzerland) was added to the corresponding volume of medium. Thus, 250 µL WST were added to the wells with sample (TCP + R), and 40 µL were added to the old wells (TCP and C+), the blank wells, and the positive control (C+). This was incubated in an incubator at 37 °C for 2 h. After this time, the liquids were transferred into a 96 well plate. Three times, 100 µL of each solution were added to the wells. The absorption was then measured at 450 nm using a Spectrostar Nano microplate reader (BMG Labtech, Ortenberg, Germany). The experiment was performed at least three times for each time point (3, 7, and 10 days).

#### 2.3.3. Lactate Dehydrogenase (LDH) Assay

The scaffolds for use in the lactate dehydrogenase LDH experiment were seeded in three 12 well plates. Each experiment assessed 3 scaffolds from each size, three Thermanox Coverslips each as controls, a positive control, a negative control, and a blank to account for background absorbance in the ELISA reader. The experiments were repeated at least three times. A 200 µL cell solution containing 50,000 cells was seeded onto each scaffold, and 100 µL cell solution containing 50,000 cells was seeded onto the Thermanox Coverslips and additionally into two empty wells to act as the positive and negative controls, respectively. One well was left empty for use as a blank. The well plate was placed in an incubator at 37 °C with 5% CO_2_ for 2 h. Following incubation, 2.5 mL of DMEM-F12 phenol red free with the 1% P/S and 1% FBS additives was added into the samples wells and negative control wells. Since FBS itself contains LDH, a concentration of 10% in the medium might have triggered background absorption. Therefore, only a concentration of 1% FBS was added to the medium. For the positive controls, 1% Triton X 100 (Art. No. X100, Sigma Aldrich, Saint Louis, MO, USA) was added to the DMEM-F12 medium with 1% P/S and 1% FBS to 100%. kill the cells, The LDH experiments were carried out at 24, 48 and 72 h following seeding and the same procedure was repeated at each interval: Three 100 µL samples were taken from each well into a 96 well plate. An LDH reagent (100 µL) was added to each well in use, and the plate was incubated in darkness at room temperature for 30 min. Following incubation, the plate was placed in a Spectrostar Nano microplate reader, and absorbance was measured at a λ of 490 nm with a reference λ of 600 nm.

### 2.4. Incubation in Simulated Body Fluid (SBF)

For the preparation of an SBF solution, 500 mL of distilled water were required, as were the chemicals described in Table 1 with quantity indication [20].

The solution was then sterilized under sterile conditions with a 0.2 µm filter. Five scaffolds of each size were placed in a 12 well plate, and each was covered with 3.5 mL of an SBF solution. The plate was then incubated for 28 days in an incubator at 37 °C with 5% CO_2_ saturation. After 28 days, the SBF solution was aspirated, and the wells filled with scaffolds were washed three times with distilled water and then dried in the drying oven at 40 °C. The wells were then incubated for 28 days at 37 °C with 5% CO_2_ saturation.

### 2.5. Statistics

Data are expressed as mean values ± standard deviation of the mean and analyzed by a one-way analysis of variance (ANOVA). The level of statistical significance was set at *p* < 0.05. For statistical calculations, Origin 2018 Professional SR1 (OriginLab, Northampton, MA, USA) was used.

## 3. Results

### 3.1. Sample Characterization

#### 3.1.1. Weight and Dimensions

**Figure 1:**
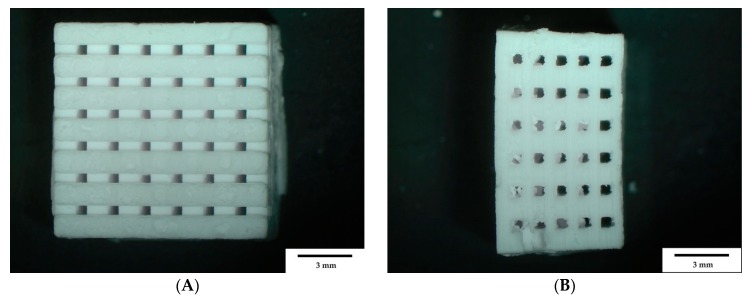
β-TCP scaffold (500 µm) through a stereomicroscope (Olympus SZ61). **A**: Top view; and **B**: Side view. Scale bar = 3mm; magnification: 0.67×.

For the characterization of the β-TCP scaffolds, these were weighed and measured once. Figure 1 shows two different views of the β-TCP scaffold, exemplary at the scaffold with the 500 µm pore size. Figure 1A shows the top view of the scaffold, and Figure 1B shows the side view of the sample.

For the scaffold dimension, at least five samples of all three scaffold sizes were measured. Table 2 shows the dimensions and weights of the β-TCP scaffolds. It can be seen that the 1000 µm scaffolds were the largest samples. The 750 and 500 µm samples were very close, with lengths of 13.05 and 12.71 mm and widths of 13.08 and 12.74 mm. 

#### 3.1.2. Surface Roughness

To determine the surface roughness, three scaffolds of each size and five different areas on the surface of each scaffold were measured. The surface roughness parameter S_a_ was averaged from all values, and these measurements are shown in Table 3. The scaffolds with a strand width of 1000 µm showed the highest roughness on the surface of 9.61 ± 2.02 µm. The 750 µm scaffolds has the lowest surface roughness of 7.97 µm ± 1.54 µm. With *p* > 0.05 the surface roughness values showed no significant differences.

#### 3.1.3. Mechanical Strengths

The values in Table 4 show that the scaffolds became more fragile as the strand width increased. The maximum failure load continued to decrease. This value was highest for the 500 µm scaffolds at 543.6 ± 35 N, while the 1000 µm scaffolds broke at a force of 117.5 ± 43.4 N. The maximum failure load was always lower at increased strand widths. The compressive strength of the specimens was then calculated. The results show that the resistance of the 500 µm scaffolds was highest at 3.4 ± 0.2 MPa. In comparison, the 1000 µm scaffolds had the lowest resistance of 0.5 ± 0.18 MPa.

In a second experiment, the scaffolds were incubated for 28 days in SBF at 37 °C. After this time, the samples were dried and also tested for maximum failure load. The following table shows the results of all scaffold sizes. In addition, the compressive strength was also calculated for the SBF-treated samples, as shown in Table 5. Again, the 500 µm scaffolds had the highest compressive strength of 2.8 ± 0.2 MPa, while the other two sizes had a compressive strength of 0.8 ± 0.1 MPa (750 µm) and 0.28 ± 0.1 MPa (1000). Incubation in SBF led to a reduction in compressive strength to 82% (500), 62% (750), and 56% (1000).

#### 3.1.4. Microstructure and Elemental Analysis

**Figure 2:**
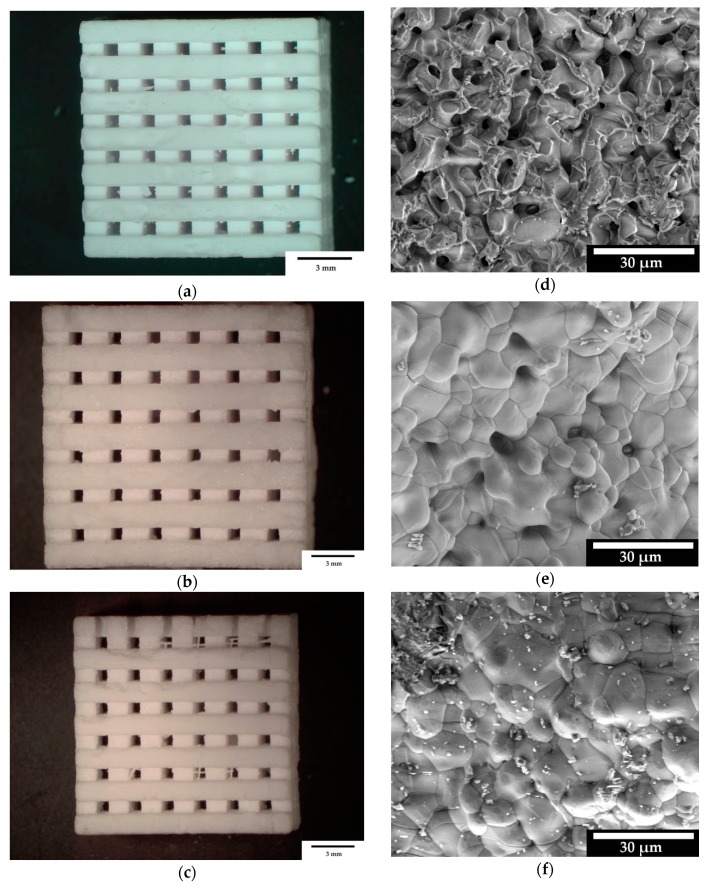
Macro and microstructure of the scaffolds; stereomicroscopic images of all three sizes: (**a**): 500 µm empty space; (**b**): 750 µm; and (**c**): 1000 µm. microstructures taken with FEI QUANTA 250 FEG, 20 kV, 3200x magnification of the surface from sample: (**d**): 500 µm; (**e**): 750 µm; and (**f**): 1000 µm.

In order to determine the microstructure of β-TCP´, images were taken with an ESEM. Figure 2 shows the microscope images of all three scaffold sizes with an HFW of 93.3 µm (right) and the measured spots on the surface of the samples (left). It can be seen that the scaffolds with a pore size of 500 µm had the highest number of micro pores. The 750 and 1000 µm samples showed that the micro pores were fused into each other and largely closed.

**Figure 3:**
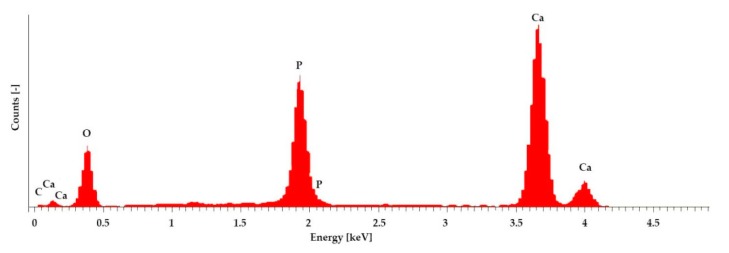
EDX spectrum of the samples, taken with an FEI Quanta ESEM 250 FEG and an Oxford EDX unit, 10 kV acceleration voltages, and 10 min counting period live time corrected.

To determine the elements in the sample, an elemental analysis of the grape-like surface structure on the various samples (see Figure S1) was performed using EDX. Figure 3 shows an example of the EDX spectrum of the 1000 µm samples. Table 6 shows the percentages for relevant atoms for all samples.

#### 3.1.5. Porosimetry

**Figure 4:**
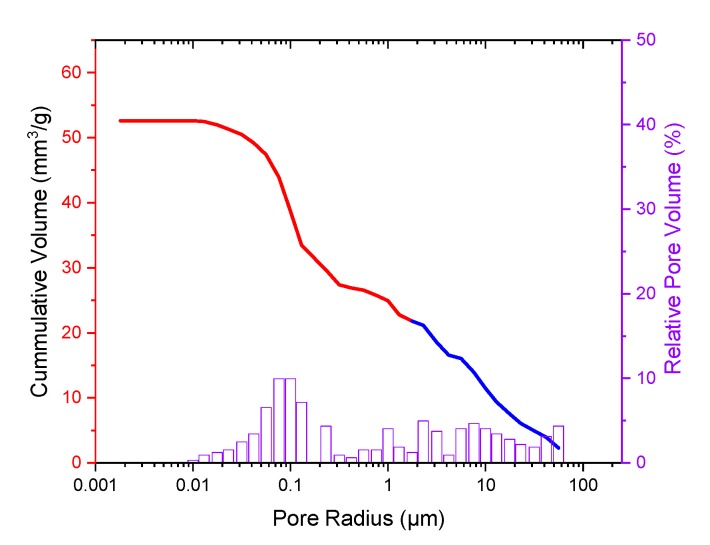
Pore size distribution of the β-TCP determined using Pascal 140 (blue curve) and 440 (red curve) mercury porosimeter, relative pore volume (%) in violet.

The pore size distribution of the micro pores was measured using a Pascal 140/440 mercury porosimeter (see Figure 4). The percentage of pores of the ceramic scaffold was 13.75%. The average pore radius was 0.086 µm.

### 3.2. Biocompatibility

#### 3.2.1. Live/Dead Assay

**Figure 5:**
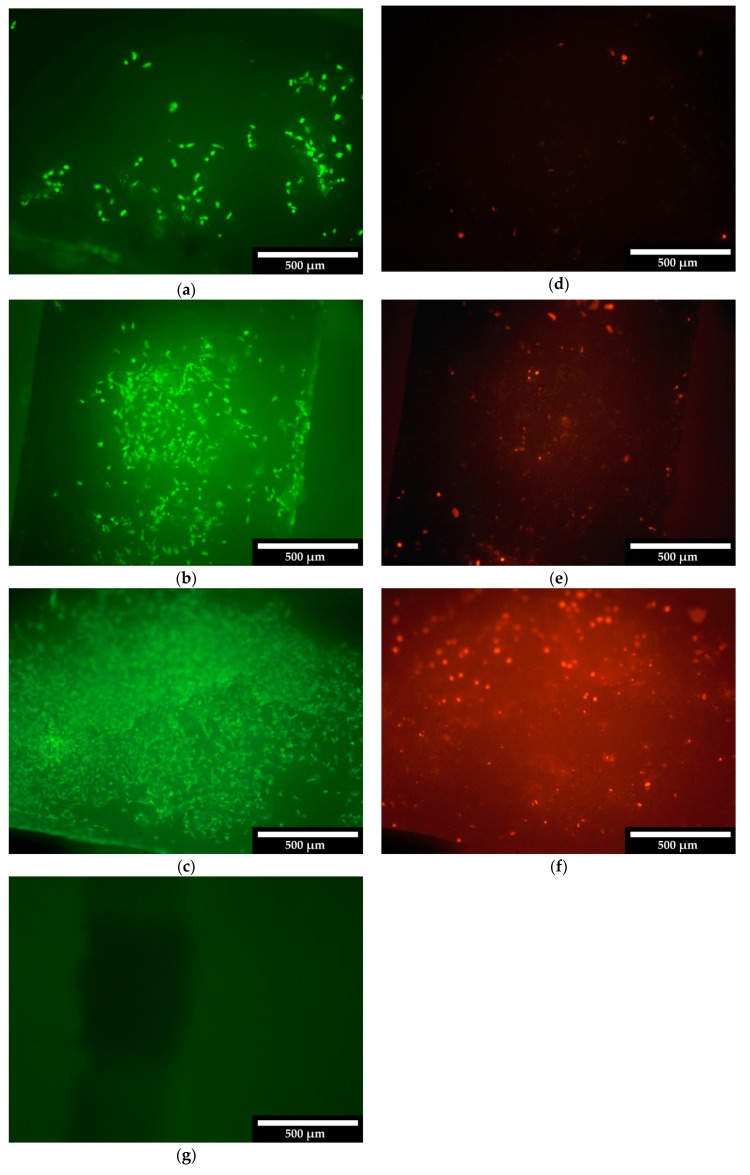
Living/dead cells on the outer surface of the ceramics, 500 µm scaffold after three days (**a**: Living; **d**: Dead), seven days (**b**: Living; **e**: Dead) and 10 days (**c**: Living; **f**: Dead); **g**: Auto-fluorescence of the ceramics; white bar = 500 µm.

The cells were counted using Image-J (Fiji), through which the cell number/mm² and the percentage of living cells were determined. Figure 5 shows the live/dead staining of the 500 µm scaffolds after 3, 7 and 10 days. Figures S2 and S3 in the supplement show the live/dead staining for the 750 µm or 1000 µm scaffolds.

**Figure 6:**
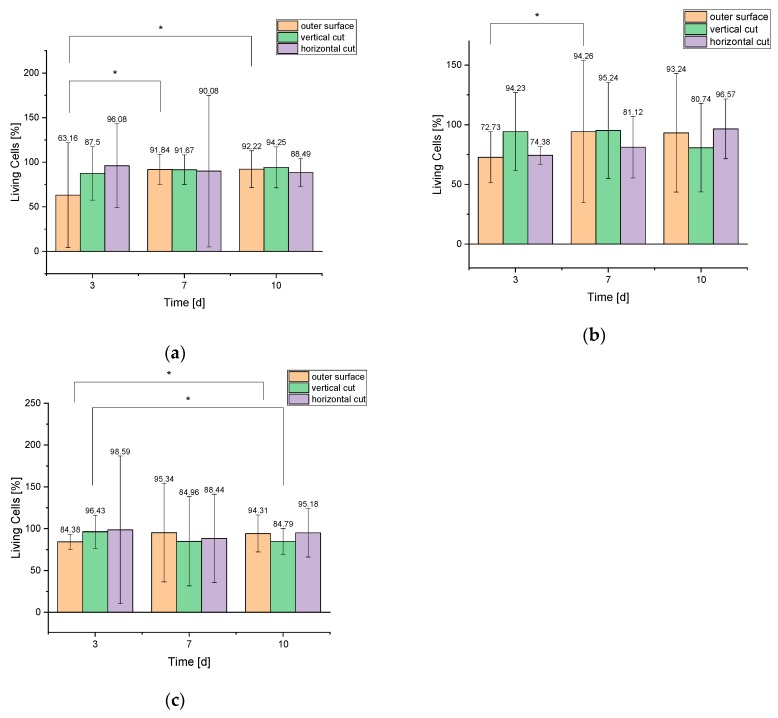
Living cells in percent for the different scaffolds **a**: 500 µm; **b**: 750 µm; and **c**: 1000 µm.

The numbers of living and dead cells per mm² over all test days are shown in Table 7. Different locations of the scaffolds (outer surface, vertical and horizontal cuts) were considered. It can be seen that the number of cells per mm² increased continuously over the course of 10 days. Figure 6 summarizes the percentage of living cells on and in the various scaffolds. 

#### 3.2.2. Cell Proliferation Assay

**Figure 7:**
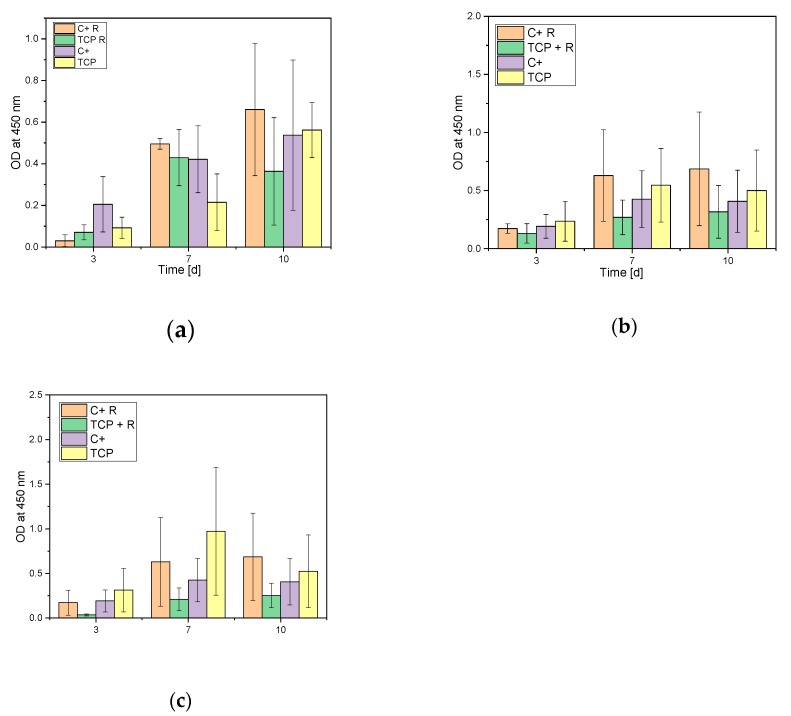
Differences in WST-I assay of MG-63 cells after cultivation on the different samples after **a**: 500; **b**: 750, and **c**: 1000; C + = Positive control/cells on Thermanox, TCP = Cells on sample, C + R = remaining cells in the control cell culture plate, TCP + R = Remaining cells in the TCP sample cell culture plate.

Figure 7 shows that the growth rate of all different macro pores sized steadily increased in all wells, whereas the growth rate of the remaining cells in the cell culture plates increased only up to seven days and stagnated thereafter.

#### 3.2.3. LDH Assay

**Figure 8:**
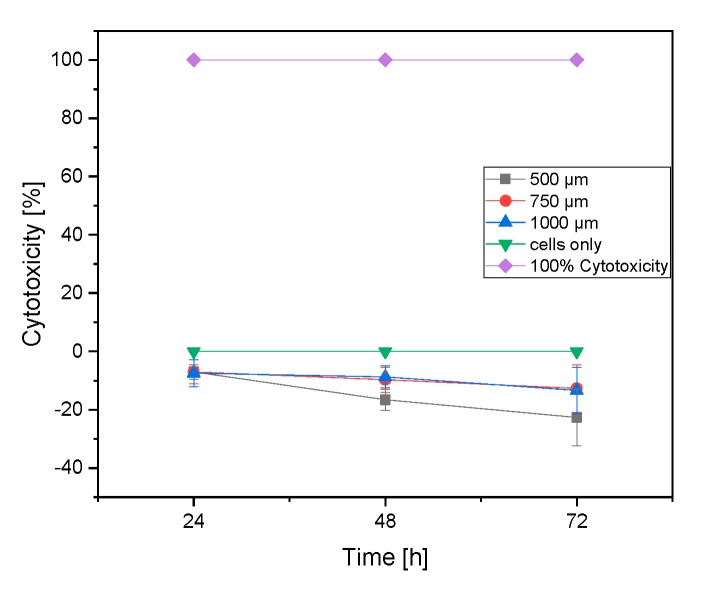
Lactate dehydrogenase (LDH) activity of MG-63 cells on the samples measured after 24, 48 and 72 h (normalized to cell control = cells only)**.**

An LDH assay was performed to determine the damaged cells on the β-TCP scaffolds. All results show negative values. These could all be regarded as 0 (no cytotoxicity at all) (see Figure 8).

## 4. Discussion

### 4.1. Mechanical Stability

The mechanical stability of the scaffolds decreased with increasing pore size. Scaffolds with a 500 µm pore size had the highest compressive strength of 3.4 ± 0.2 MPa and scaffolds with a 1000 µm pore size the lowest of 0.5 ± 0.18 MPa. Bose et al. [21] investigated the mechanical properties of cylindrical TCP scaffolds with different pore volumes and strand spacings (voids); their results showed that the compressive strength of the samples decreased with increasing pore volumes. Bose et al. [21] worked with pore volumes between 29% and 44%; in this project, a pore content of 13.75% was measured. Looking at the compressive strength results of both projects, it can be seen that the samples in this project had a compressive strength of 3.4 ± 0.2 (500) or 2.8 ± 0.2 MPa (500 + SBF) than the samples in the project of Bose et al. [21]. Here, the compressive strength of comparable scaffold sizes was 0.2 MPa. With a compressive strength of 3.4 ± 0.2 (500) or 2.8 ± 0.2 MPa (500 + SBF), as in the present project, one comes close to the stability of a cancellous bone with a compressive strength in the range of 2–20 MPa [22]. However, the stability of the scaffolds was still too low for cortical bone tissue structure. Here, the compressive strength lied in a range of 100–200 MPa [22]. If one compares the results of the compression test of the present project with the results of our previous work [7], which also dealt with 3D printed β-TCP scaffolds, lower values for the powder-printed cylindrical scaffolds can be found. The scaffolds of our previous work [7] withstood a force of only 54.3 ± 14.5 N and had a compressive strength of 0.64 ± 0.17 MPa. However, our previous work had another question in the foreground. Sophisticated structures that depend on the grain size of the ceramic powder were investigated. In contrast to the present work, the scaffolds were printed by a 3D powder printing process in our previous work.

### 4.2. Sample Characterization via Porosimetry, ESEM and EDX

The ESEM images in Figure 2 show that the scaffolds with a pore diameter of 500 µm had the highest micro pore content within the ceramic compared to those of 750 and 1000 µm. This result was not expected. According to Vorndran et al. [23], scaffolds with well-connected pores have a higher stability than those with larger pores that are less interconnected. However, this result shows that the micro pore structure within the ceramic alone was not the decisive factor for the stability of the scaffolds. 

The ESEM images of the scaffolds previously placed in an SBF solution show hydroxyapatite crystallization on the surface of the 750 and 1000 µm strand spacing samples (see Figure S1). The crystals did not show up as project needle-shaped (as in our previous work [8]), instead showing up in a grape-like form. This crystal form was also investigated in the project of Lei et al. [24]. Here, a composite of polycaprolactone (PCL) and β-TCP was used instead of pure β-TCP scaffolds. 

An EDX analysis was performed for a clear detection of hydroxyapatite HA. The X-ray spectrum in Figure 3 and the values in Table 6 show that calcium and phosphate were present in the sample in a ratio of 1.68, and HA was thus proven [5,25,26,27].

### 4.3. Biocompatibility

#### 4.3.1. Live/Dead Assay

All three scaffold sizes showed an increase in cell count. Figure 5 shows the cell growth on the surface of the 500 µm scaffolds. Figure S2 shows the growth behavior of the cells within the 750 µm and Figure S3 within the 1000 µm scaffolds. If one compares the growth on the scaffolds with the growth of the cells within the scaffolds, it becomes clear that most of the cells adhered to the outer surface. The reason for this is that the cells for cultivation on the samples were placed on the surface via a pipette and therefore largely adhered there. In addition, the scaffolds were hygroscopic, i.e., all liquid with the cells was completely absorbed.

#### 4.3.2. Cell Proliferation Assay (WST-I)

With few exceptions, the proliferation rate of MG-63 cells steadily increased during the test days. The proliferation rate on the 500 µm scaffolds increased from day three to day 10 by approximately 130% (see Figure 7). The absorption rate in the empty wells also increased for all three samples over the 10 test days, which shows that the cells also migrated through the samples and adhered to the bottom of the microtiter plate. However, the values were much lower than in the wells with the samples. The reason for this is that the cells mainly remained on and inside the scaffolds, and only a few migrated through the sample. If one compares the results with a previous paper [8], it can be seen that an increasing proliferation over the 10 test days on the scaffolds was also achieved there. However, the rate in the empty samples of wells was higher than in this project. The reason for this can be determined by finding the amount of cells that migrated through the sample and did not remain on or within the scaffolds. This can be explained by the geometry of the samples. In the previous work [7], we worked with cylindrical scaffolds whose voids ran only in one direction and which has no other internal structures. Thus, the liquid with the cells penetrated completely through the scaffold to the bottom, which is why high absorption values were measured in the empty wells. In this project, the focus was on the inner structure of the scaffolds, which was why the scaffolds were hygroscopic and most of the liquid remained in the sample instead of flowing through it.

#### 4.3.3. LDH Assay

The LDH test showed that the cells showed no LDH activity, and the β-TCP therefore did not show any cytotoxicity. The results were in a negative range, so they could be considered 0. β-TCP is an already approved biomaterial for bone replacement [5], which is why this result was expected.

## 5. Conclusions

It has been proven that different strand distances (voids) influence the stability of β-TCP scaffolds. The 500 µm scaffolds have the highest compressive strength of all three sizes due to them having the smallest voids. They have the stability of a cancellous bone. The 500 µm scaffolds are therefore best suited for use in bone replacement. However, the variation of the strand spacing (voids) has no influence on biocompatibility. Almost identical cell growth was observed for all three sizes. The cell growth behavior within the β-TCP scaffolds and the biocompatibility of the material were also successfully demonstrated. A large number of the cells grew into the scaffold and were not only adhered to the surface of the samples. Thus, it could be proven that the method of inverse 3D pressure is excellently suited for the ingrowth of cells into scaffolds. The biocompatibility of β-TCP was also successfully tested. The cells showed a high proliferation rate on the scaffolds, and no cytotoxicity was measured from the material. In addition, the degradation of the material, which is important for bone replacement, could be demonstrated with the help of simulated body fluid. β-TCP showed an incipient degradation of the material after a 28-day incubation in an SBF solution, which could be detected by the formation of HA crystals on the samples. In conclusion, it can be said that β-TCP is biocompatible and thus a suitable material, and the inverse FDM printing process with subsequent slip casting is a suitable method for use in bone replacement.

## Figures and Tables

**Table 1:** Simulated body fluid (SBF) receipt [20].

Chemical Substance	Quantity [g]
NaCl	3.274
NaHCO_3_	1.1134
KCl	0.187
Na_2_HPO_4_ 2H_2_O	0.089
MgCl_2_	0.071
CaCl_2_ 2H_2_O	0.184
Na_2_SO_4_	0.0355
(CH2OH)_3_CNH_2_	3.0285
1M HCl solution	until pH 7.4

**Table 2:** Sample dimensions and weights.

Sample	Length (mm)	Width (mm)	Height (mm)	Weight (g)
500 µm	12.71 ± 0.05	12.74 ± 0.14	5.59 ± 0.04	1.48 ± 0.09
750 µm	13.05 ± 0.34	13.08 ± 0.2	6.43 ± 0.17	1.63 ± 0.17
1000 µm	14.67 ± 0.53	14.65 ± 0.10	8.13 ± 0.15	2.38 ± 0.12

**Table 3:** Measurement results of surface roughness with n = 15 and a 400x magnification.

-	Sa (µm)		
**Material**	500 µm	750 µm	1000 µm
**Mean ± SD**	8.84 ± 0.71	7.97 ± 1.54	9.61 ± 2.02

**Table 4:** Breaking and compressive strength of the different sized samples (N = 10)**.**

500 µm			750 µm			1000 µm		
Breaking Strength [N]	F_max_ [N]	Compressive Strength [MPa]	Breaking Strength [N]	F_max_ [N]	Compressive Strength [MPa]	Breaking Strength [N]	F_max_ [N]	Compressive Strength [MPa]
543.6 ± 35	2754.6 ± 234.9	3.4 ± 0.2	243.4 ± 34.1	1210.6 ± 146.7	1.3 ± 0.2	117.5 ± 43.4	309 ± 89.1	0.5 ± 0.18

**Table 5:** Breaking and compressive strength of the different SBF-treated samples (N = 10)**.**

500 µm + SBF			750 µm + SBF			1000 µm + SBF		
Breaking Strength [N]	F_max_ [N]	Compressive Strength [MPa]	Breaking Strength [N]	F_max_ [N]	Compressive Strength [MPa]	Breaking Strength [N]	F_max_ [N]	Compressive Strength [MPa]
458.6 ± 40.2	2094.8 ± 712.4	2.8 ± 0.2	155.3 ± 21.3	786.4 ± 104.9	0.8 ± 0.1	64 ± 24	150 ± 61	0.28 ± 0.1

**Table 6:** Proportions of relevant elements in the EDX spectrum.

Elements	Atom [%]
C	7.79
O	64.83
P	9.85
Ca	16.53

**Table 7:** Overview of living/dead cells for the different sized scaffolds.

-	Day 3		Day 7		Day 10	
	Living	Dead	Living	Dead	Living	Dead
**500 µm**	**Cells/mm²**					
**Outer surface**	36 ± 33	21 ± 1	180 ± 33	16 ± 8	308 ± 69	26 ± 6
**Vertical cut**	35 ± 12	5 ± 4	121 ± 20	11 ± 8	164 ± 40	10 ± 4
**Horizontal cut**	49 ± 24	2 ± 1	109 ± 115	12 ± 13	123 ± 22	16 ± 11
**750 µm**	-	-	-	-	-	-
**Outer surface**	32 ± 9	12 ± 4	197 ± 124	12 ± 8	262 ± 139	19 ± 8
**Vertical cut**	49 ± 17	3 ± 2	200 ± 84	10 ± 4	218 ± 99	52 ± 23
**Horizontal cut**	119 ± 9	2 ± 2	189 ± 59	44 ± 21	225 ± 58	8 ± 2
**1000 µm**	-	-	-	-	-	-
**Outer surface**	27 ± 2	5 ± 5	225 ± 139	11 ± 9	265 ± 61	16 ± 11
**Vertical cut**	54 ± 11	2 ± 1	226 ± 141	40 ± 22	223 ± 44	40 ± 11
**Horizontal cut**	70 ± 84	1 ± 2	176 ± 105	23 ± 38	158 ± 48	8 ± 0

## Supplementary Materials

The following are available online at https://www.mdpi.com/1996-1944/12/20/3417/s1, Figure S1. SBF treated samples, left = ESEM images of all three scaffold sizes (a: 500 µm; b:750 µm; c: 1000 µm) with HFW of 93.3 µm; right = magnification of selected section with HFW of 11.7 µm (d: 500 µm; e: 750 µm; f: 1000 µm ), Figure S2: Living/Dead Cells inside the ceramics, vertical cut of 750 µm scaffold after three days (a: Living; d: Dead), seven days (b: Living; e: Dead), and 10 days (c: Living; f: Dead); white bar = 500 µm, Figure S3: Living/dead Cells on top of the 1000 µm scaffold after 3 days (a: living; d: dead), 7 days (b: living; e: dead) and 10 days (c: living; f: dead); g: auto-fluorescence of the ceramics; white bar = 500 µm.
