# Supplementary material for: Inversely 3D-Printed β-TCP Scaffolds for Bone Replacement
Source: Materials (Basel). 2019 Oct 18;12(20):3417. doi: 10.3390/ma12203417 (PMC6829219; doi:10.3390/ma12203417)
Supplement: Supplementary file 1 [file materials-12-03417-s001.pdf]

materials

Supplementary Materials

Inversely 3D-Printed β-TCP Scaffolds for Bone Replacement

Michael Seidenstuecker [1,*], Svenja Lange [1,2], Steffen Esslinger [3,4], Sergio H. Latorre [1], Rumen Krastev [2], Rainer Gadow [3,4], Hermann O. Mayr [1] and Anke Bernstein [1]

1. G.E.R.N. Tissue Replacement, Regeneration & Neogenesis, Department of Orthopedics and Trauma Surgery, Medical Center - Albert-Ludwigs-University of Freiburg, Faculty of Medicine, Albert-Ludwigs-University of Freiburg, Hugstetter Straße 55, 79106 Freiburg, Germany
2. Faculty of Applied Chemistry, Reutlingen University, Alteburgstraße 150, 72762 Reutlingen, Germany
3. Institute for Manufacturing Technologies of Ceramic Components and Composites (IMTCCC), Faculty 07, University of Stuttgart, Allmandring 7b, 70569 Stuttgart, Germany
4. GSaME – Graduate School of Excellence advanced Manufacturing Engineering; University of Stuttgart, Nobelstraße 12; 70569 Stuttgart; Germany

* Correspondence: michael.seidenstuecker@uniklinik-freiburg.de; Tel.: +49-761-270-26104

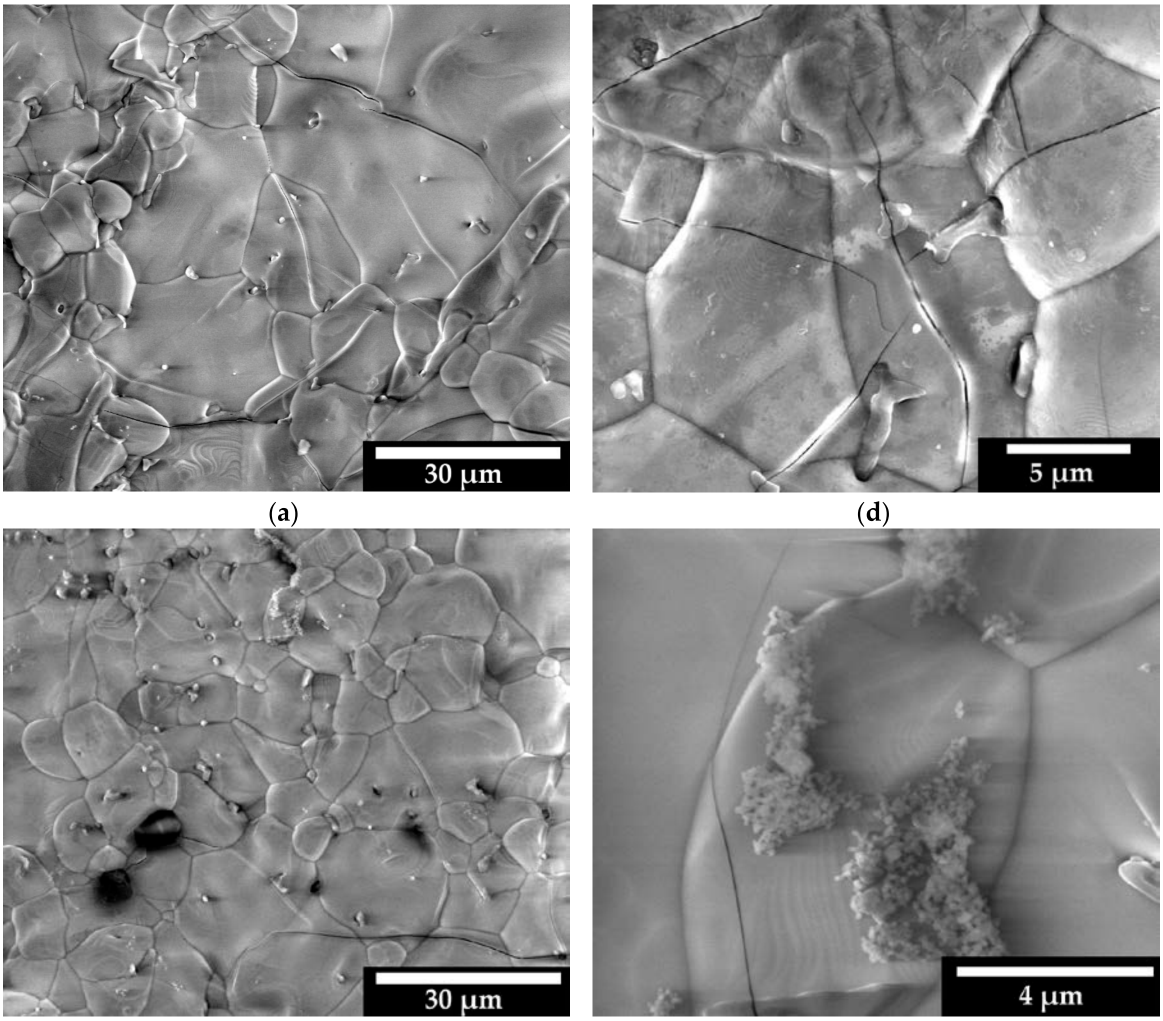

(**a**) (**d**)

(**b**) (**e**)

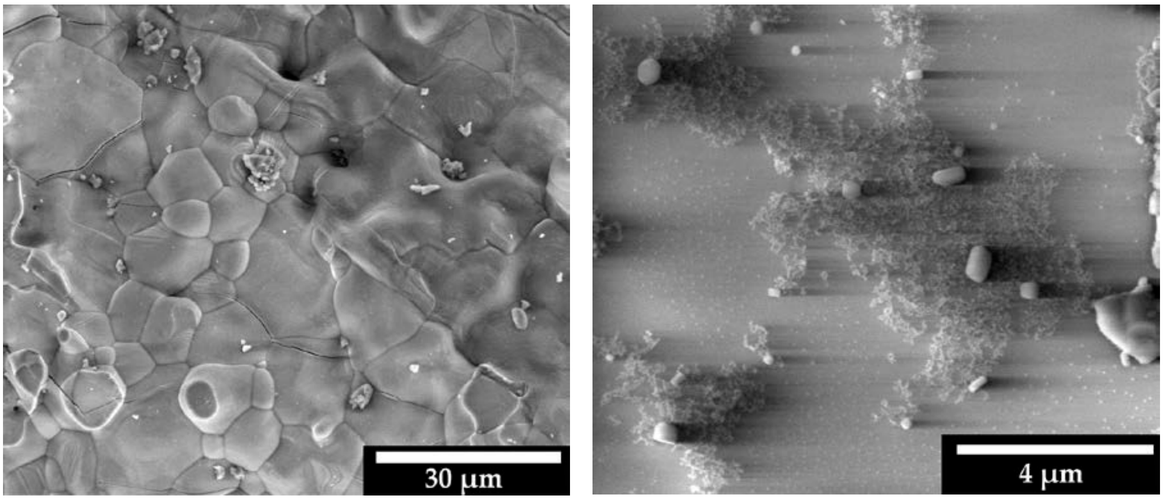

(**c**) (**f**)

Figure S1. SBF treated samples, left = ESEM images of all three scaffold sizes (**a**: 500 µm; **b**:750 µm; **c**: 1000 µm) with HFW of 93.3 µm; right = magnification of selected section with HFW of 11.7 µm (**d**: 500 µm; **e**: 750 µm; **f**: 1000 µm)**.**

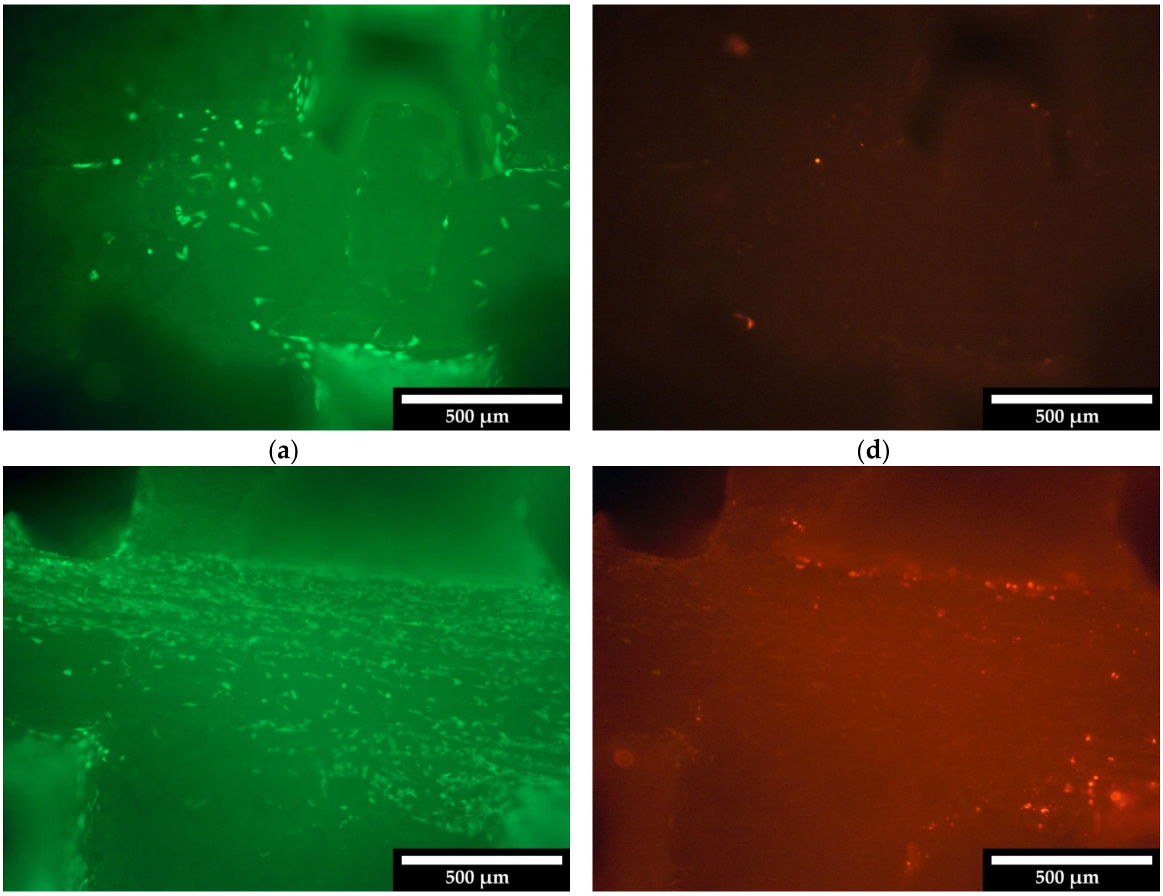

(**b**) (**e**)

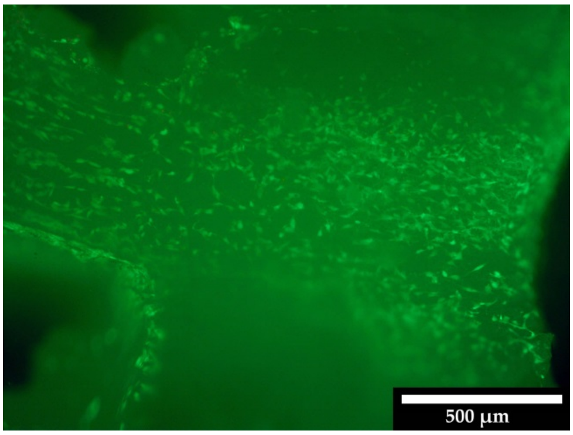

(**c**)

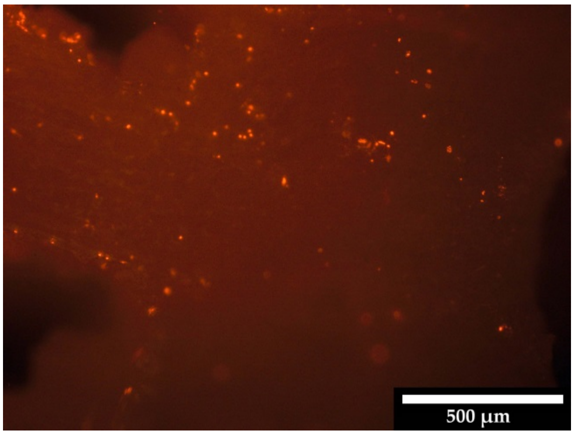

(**f**)

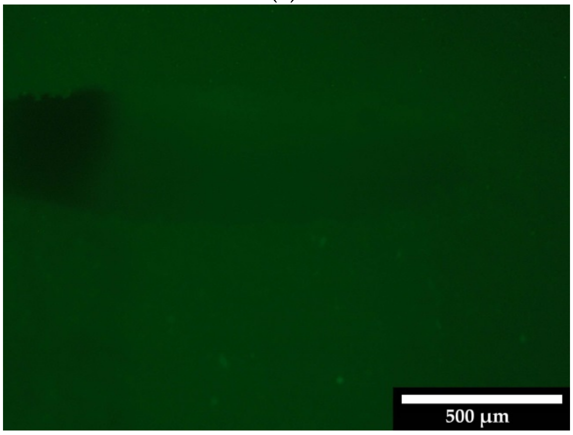

(**g**)

Figure S2. Living/dead Cells inside the ceramics, vertical cut of 750 µm scaffold after 3 days (**a**: living; **d**: dead), 7 days (**b**: living; **e**: dead) and 10 days (**c**: living; **f**: dead); **g**: auto-fluorescence of the ceramics; white bar = 500 µm.

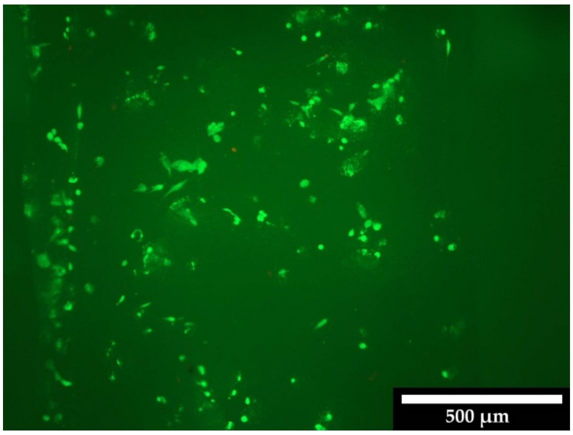

(**a**)

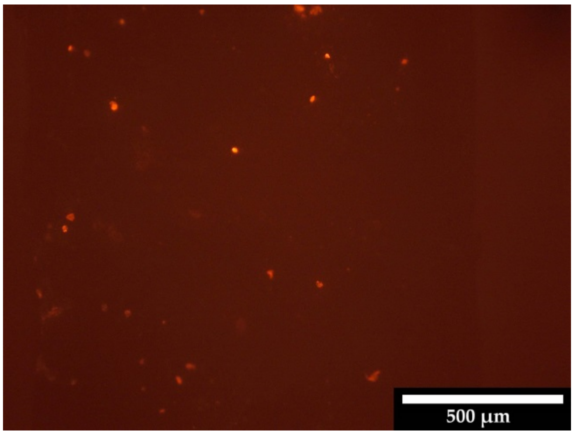

(**d**)

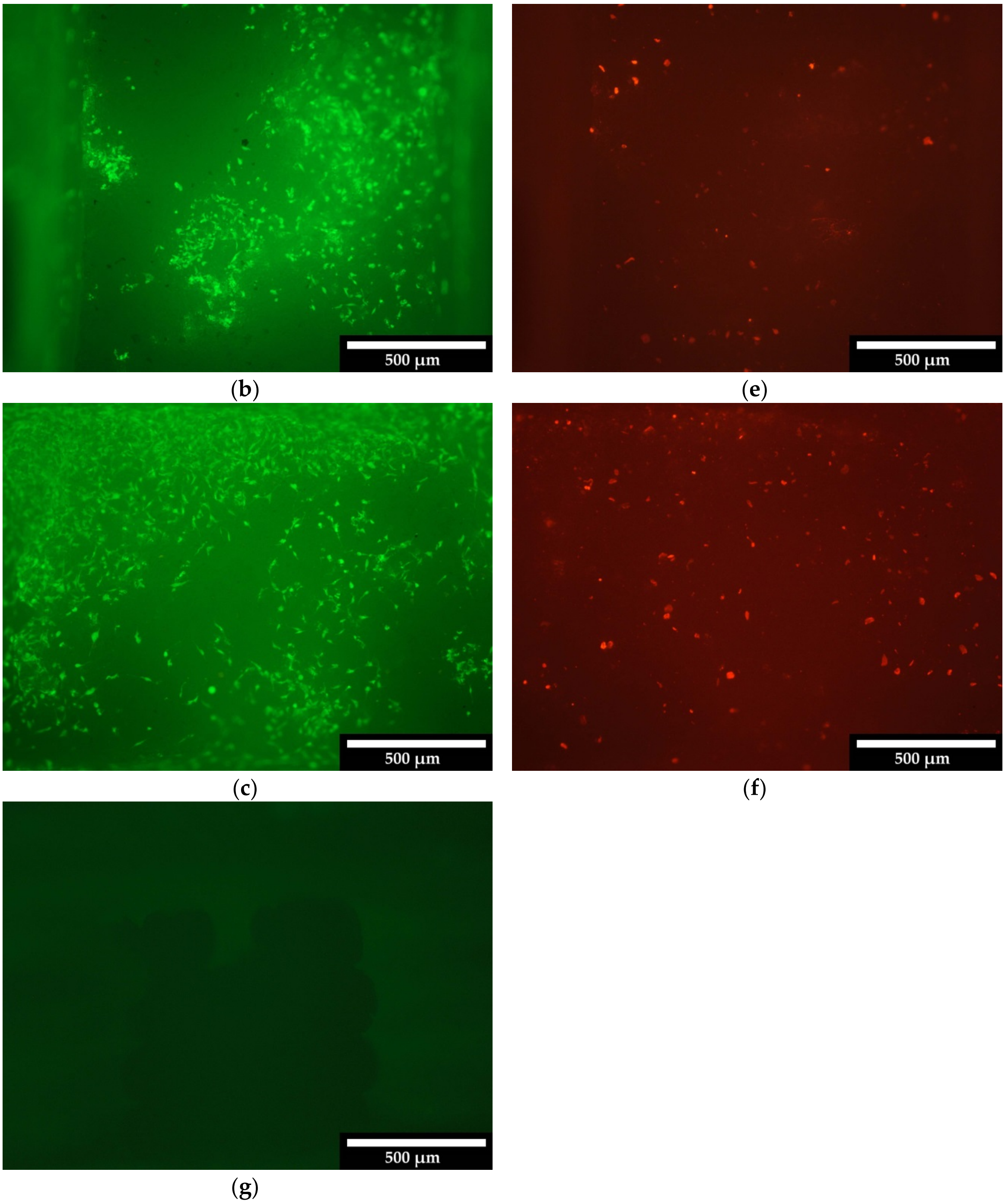

Figure S3. Living/dead Cells on top of the 1000 µm scaffold after 3 days (**a**: living; **d**: dead), 7 days (**b**: living; **e**: dead) and 10 days (**c**: living; **f**: dead); **g**: auto-fluorescence of the ceramics; white bar = 500 µm.
